# Supplementary material for: Automatic variable selection in ecological niche modeling: A case study using Cassin’s Sparrow (Peucaea cassinii)
Source: PLoS One. 2022 Jan 21;17(1):e0257502. doi: 10.1371/journal.pone.0257502 (PMC8782318; doi:10.1371/journal.pone.0257502)
Supplement: S1 Appendix — Overview of MERRA/Max’s default screening parameters and supporting documentation. (PDF) [file pone.0257502.s001.pdf]

APPENDIX

MERRA/Max Parameterization

We envision MERRA/Max being used as a high-performance discovery tool: a means of screening a large or unfamiliar collection of environmental variables for a set of predictors that subsequently may be ignored, modified, or employed in the construction of an ecological niche model. Since *a priori* assumptions cannot be made about MERRA/Max's inputs or outputs, we adopted a standard screening configuration for several parameters that control a MERRA/Max run and used these as defaults in all our timing trials and use case scenarios. This appendix describes MERRA/Max's default parameter settings and the rationale behind the choices we made. While these choices were guided by data and experience, they are ultimately somewhat arbitrary. We look forward to further refinement and formalization of the approach.

MaxEnt settings

We used a MaxEnt feature class (FC) setting of LQHP (linear, quadratic, hinge, and product) and a regularization multiplier (RM) setting of 1.0 in MERRA/Max's selection runs. These choices were based on an ENMeval [1,2] scan of the 19 Bioclim variables over the study area in which the lowest AICc and best overall model resulted from these settings (Table 1).

Sampling sizes

MERRA/Max's run time performance and selection behavior are potentially influenced to by two types of sampling: the average number of random samples taken of each variable in the collection being scanned (S) and the number of random variables used in each sprint's independent MaxEnt sampling run (V). In our default screening configuration, we used S = 50 and V = 2 throughout. We arrived at these choices by a qualitative assessment of MERRA/Max's overall behavior in runs using our development test data, which comprised 2016 Cassin's Sparrow observations and the 19 Bioclim predictors. These results are summarized in Tables 2 and 3.

For reference, Table 2 shows values of the Pearson correlation coefficient (r), coefficient of determination (r^2), and variance inflation factor (VIF) [$1 \div (1 - r^2)$] for the Worldclim Bioclimatic variables over our study area [3]. Values of $r > 0.8$, $r^2 > 0.8$, and VIF > 10.0 are highlighted and indicate highly correlated variables. Table 3 shows the results we obtained in ensembles where the average number of random samples for each collection variable was S = 50 (Table 3a), S = 100 (Table 3b), and S = 150 (Table 3c). Since we were using the Bioclim dataset, the collection size (N) was 19 throughout. For each value of S, runs were performed on values of V ranging from two to 19. Color-coded columns on the left in Table 3 show the various configurations of virtual machines, processor cores, and sprint runs needed to achieve the desired V and S sampling rates.

MERRA/Max's selection behavior across the various ensemble configurations is color-coded against Table 2's correlation analysis. Selected variables for which little or no correlation exists are indicted in green text. Variables that have high correlation with one or more other variables in the selection set are indicated in red text. Variables contributing to an optimal non-correlated selection set are highlighted by a green background. These correspond to the set of variables identified in the accompanying paper's use case scenarios after the *Predictor Refinement* step. The model performance metrics associated with each ensemble reflect the results of a final MaxEnt model run using the post-refinement selection set. Generally speaking, there appears to be no significant improvement in overall selection performance with values of S > 50 or V > 2. For the current study, we chose S = 50 and V = 2 as default values for MERRA/Max, because these settings result in the shortest run times.

Selection criterion

MaxEnt provides three algorithm-specific indicators of variable importance: percent contribution (PC), permutation importance (PI), and change-in-gains based on training and test jackknife analyses of individual variables (JK) [4-6]. No single measure is sufficient to identify which variables are best for producing a final model [7]. However, in an exhaustive comparison of MaxEnt models resulting from all pairwise combinations of Bioclim's 19 variables, we observed only 10 instances where permutation importance did not agree with the other indicators (Table 4, highlighted in blue). For MERRA/Max's screening purposes, we felt comfortable using permutation importance as our sole indicator of variable importance.

Appendix accompanying Schnase et al. (2022). *Automatic variable selection in ecological niche modeling*.

Table 1. ENMeval feature class and regularization multiplier scan.

	settings	features	rm	train.AUC	avg.test.AUC	var.test.AUC	avg.diff.AUC	var.diff.AUC	avg.test.orMTP	var.test.orMTP	avg.test.or10pct	var.test.or10pct	AICc	delta.AICc	w.AIC	parameters
1	L_0.5	L	0.5	0.5554	0.522482743	0.009828229	0.039713398	0.009681591	0.003278689	4.78E-05	0.116693989	0.00180239	12909.84862	378.1636922	2.74E-83	14
2	LQ_0.5	LQ	0.5	0.6081	0.580776749	0.009491528	0.034170297	0.007958198	0.003278689	4.78E-05	0.124754098	0.001256801	12630.90673	99.22180154	1.02E-22	22
3	H_0.5	H	0.5	0.6534	0.574461093	0.008691308	0.082685676	0.009088654	0.024617486	0.00055193	0.149398907	0.002526879	15060.43947	2528.754543	0	84
4	LQH_0.5	LQH	0.5	0.6534	0.574463716	0.008691307	0.082757013	0.009136991	0.022978142	0.000429601	0.152677596	0.003098609	15060.43947	2528.754543	0	84
5	LQHP_0.5	LQHP	0.5	0.6534	0.575431619	0.008586036	0.082660917	0.00878169	0.022978142	0.000429601	0.156010929	0.003006709	15060.43947	2528.754543	0	84
6	LQHPT_0.5	LQHPT	0.5	0.6534	0.575431619	0.008586036	0.082660917	0.00878169	0.022978142	0.000429601	0.156010929	0.003006709	15060.43947	2528.754543	0	84
7	L_1	L	1	0.5316	0.519117989	0.0114877	0.025323948	0.007198028	0.001639344	2.69E-05	0.108360656	0.001322494	12954.43936	422.7544255	5.69E-93	5
8	LQ_1	LQ	1	0.5386	0.519075697	0.009618943	0.026544631	0.007676098	0.001639344	2.69E-05	0.110054645	0.000551305	12923.85092	392.1659925	2.50E-86	8
9	H_1	H	1	0.6224	0.585894301	0.008094387	0.041890321	0.006606099	0.009863388	0.000131792	0.134535519	0.001643644	12553.31931	21.63437591	7.20E-06	43
10	LQH_1	LQH	1	0.6224	0.582014587	0.008516185	0.045654071	0.007311879	0.009863388	0.000131792	0.132923497	0.00144494	12553.31931	21.63437591	7.20E-06	43
11	LQHP_1	**LQHP**	**1**	0.6258	0.588140669	0.006360179	0.041744259	0.005955404	0.006584699	7.23E-05	0.139590164	0.000676946	12531.68493	0	0.359269081	53
12	LQHPT_1	LQHPT	1	0.6258	0.588140669	0.006360179	0.041744259	0.005955404	0.006584699	7.23E-05	0.139590164	0.000676946	12531.68493	0	0.359269081	53
13	L_1.5	L	1.5	0.5325	0.516751071	0.010887471	0.026851351	0.007497633	0.001639344	2.69E-05	0.110027322	0.001319306	12947.14068	415.4557454	2.19E-91	6
14	LQ_1.5	LQ	1.5	0.5633	0.531828973	0.010733057	0.037460895	0.009162646	0.004918033	6.27E-05	0.118360656	0.001778375	12889.56966	357.8847293	6.95E-79	13
15	H_1.5	H	1.5	0.6298	0.581878941	0.009588108	0.053792812	0.008996582	0.009836066	0.000191108	0.129617486	0.001743378	12559.71114	28.02620478	2.95E-07	65
16	LQH_1.5	LQH	1.5	0.63	0.580377563	0.009796933	0.055760035	0.009257052	0.009836066	0.000191108	0.132978142	0.001154277	12559.94757	28.26263892	2.62E-07	67
17	LQHP_1.5	LQHP	1.5	0.6352	0.588544631	0.00763299	0.050983237	0.007649936	0.008224044	0.000134878	0.142868852	0.0012007	12542.29129	10.6063592	0.001787632	82
18	LQHPT_1.5	LQHPT	1.5	0.6352	0.588544631	0.00763299	0.050983237	0.007649936	0.008224044	0.000134878	0.142868852	0.0012007	12542.29129	10.6063592	0.001787632	82
19	L_2	L	2	0.5355	0.512876272	0.008943096	0.029798219	0.007475742	0.003278689	4.78E-05	0.110081967	0.00139778	12943.07993	411.3949973	1.67E-90	9
20	LQ_2	LQ	2	0.594	0.561860232	0.012019685	0.038344021	0.010739998	0.009836066	0.000131386	0.134726776	0.001632086	12794.9618	263.2768695	2.43E-58	19
21	H_2	H	2	0.6374	0.5817565	0.011049622	0.060089436	0.011949594	0.011502732	0.000182455	0.13295082	0.001746514	12630.5671	98.88216803	1.21E-22	105
22	LQH_2	LQH	2	0.6373	0.579805227	0.010685547	0.062316943	0.011881113	0.013142077	0.000286868	0.134562842	0.001823671	12614.22909	82.54415771	4.28E-19	101
23	LQHP_2	LQHP	2	0.6416	0.589332019	0.008350795	0.056263105	0.009613704	0.006557377	0.000131386	0.142868852	0.00173819	12593.83914	62.15420454	1.15E-14	104
24	LQHPT_2	LQHPT	2	0.6416	0.589332019	0.008350795	0.056263105	0.009613704	0.006557377	0.000131386	0.142868852	0.00173819	12593.83914	62.15420454	1.15E-14	104
25	L_2.5	L	2.5	0.5314	0.51986548	0.01098761	0.024121645	0.006649944	0.001639344	2.69E-05	0.105081967	0.001204246	12963.49428	431.8093446	6.15E-95	4
26	LQ_2.5	LQ	2.5	0.5384	0.518569079	0.010109175	0.026522695	0.008133702	0.001639344	2.69E-05	0.108415301	0.000799944	12938.19492	406.5099897	1.92E-89	7
27	H_2.5	H	2.5	0.6145	0.583801325	0.008053506	0.034390974	0.004395374	0.008224044	0.000134878	0.118114754	0.002006099	12585.27137	53.58643851	8.30E-13	37
28	LQH_2.5	LQH	2.5	0.6139	0.580078851	0.006600679	0.036158993	0.004856193	0.009863388	0.000251234	0.123032787	0.001673751	12599.58653	67.90159495	6.47E-16	43
29	LQHP_2.5	LQHP	2.5	0.6195	0.588158124	0.006464785	0.036226748	0.004794228	0.006584699	7.23E-05	0.136284153	0.001016334	12533.58505	1.900119329	0.138935802	34
30	LQHPT_2.5	LQHPT	2.5	0.6195	0.588158124	0.006464785	0.036226748	0.004794228	0.006584699	7.23E-05	0.136284153	0.001016334	12533.58505	1.900119329	0.138935802	34
31	L_3	L	3	0.5316	0.519156093	0.010990881	0.023886928	0.006626526	0.001639344	2.69E-05	0.11	0.001850428	12973.87437	442.1894377	3.43E-97	4
32	LQ_3	LQ	3	0.5348	0.516937836	0.010543841	0.026394864	0.008405782	0.001639344	2.69E-05	0.108387978	0.00073175	12959.23623	427.5512941	5.17E-94	8
33	H_3	H	3	0.6006	0.579181316	0.010522213	0.033655611	0.004797368	0.009863388	0.000251234	0.109918033	0.002082243	12610.01893	78.33399674	3.51E-18	29
34	LQH_3	LQH	3	0.6005	0.573521337	0.007391711	0.03517167	0.00504392	0.009863388	0.000251234	0.111557377	0.002007293	12628.08696	96.40202637	4.19E-22	37
35	LQHP_3	LQHP	3	0.6154	0.585193601	0.006519249	0.035336493	0.004353288	0.004945355	6.34E-05	0.13136612	0.000956015	12569.4178	37.73287106	2.30E-09	33
36	LQHPT_3	LQHPT	3	0.6154	0.585193601	0.006519249	0.035336493	0.004353288	0.004945355	6.34E-05	0.13136612	0.000956015	12569.4178	37.73287106	2.30E-09	33
37	L_3.5	L	3.5	0.5281	0.517386051	0.011007286	0.023488653	0.006758943	0.001639344	2.69E-05	0.119863388	0.001972058	12988.16837	456.48344	2.70E-100	5
38	LQ_3.5	LQ	3.5	0.5313	0.515441617	0.010774738	0.025728812	0.008348468	0.001639344	2.69E-05	0.113306011	0.000625148	12970.63517	438.9502417	1.73E-96	7
39	H_3.5	H	3.5	0.5957	0.57851574	0.011628851	0.031165313	0.005295744	0.008224044	0.000134878	0.111557377	0.001648967	12648.28764	116.6027045	1.72E-26	30
40	LQH_3.5	LQH	3.5	0.5957	0.571555574	0.007249283	0.033237646	0.004724316	0.008224044	0.000134878	0.114836066	0.001540872	12648.28764	116.6027045	1.72E-26	30
41	LQHP_3.5	LQHP	3.5	0.6076	0.580722522	0.005937593	0.033587728	0.00425961	0.006584699	7.23E-05	0.128060109	0.001296712	12598.45908	66.77414938	1.14E-15	25
42	LQHPT_3.5	LQHPT	3.5	0.6076	0.580722522	0.005937593	0.033587728	0.00425961	0.006584699	7.23E-05	0.128060109	0.001296712	12598.45908	66.77414938	1.14E-15	25
43	L_4	L	4	0.5268	0.517608279	0.011173645	0.022851529	0.006605521	0.001639344	2.69E-05	0.118224044	0.002136988	12994.99745	463.3125205	8.88E-102	4
44	LQ_4	LQ	4	0.5291	0.516577989	0.011450211	0.024419781	0.008059042	0.001639344	2.69E-05	0.118224044	0.001121729	12977.86017	446.1752352	4.67E-98	5
45	H_4	H	4	0.5938	0.57368697	0.011127439	0.030878127	0.006216059	0.008224044	0.000134878	0.11647541	0.001985495	12682.34061	150.6556798	6.93E-34	28
46	LQH_4	LQH	4	0.5938	0.568205911	0.007831907	0.032160881	0.005286664	0.008224044	0.000134878	0.11647541	0.001866053	12681.68317	149.9982406	9.63E-34	28
47	LQHP_4	LQHP	4	0.5996	0.577382056	0.005269511	0.030055737	0.003849446	0.008224044	0.000134878	0.12147541	0.001616322	12643.47975	111.794819	1.90E-25	25
48	LQHPT_4	LQHPT	4	0.5996	0.577382056	0.005269511	0.030055737	0.003849446	0.008224044	0.000134878	0.12147541	0.001616322	12643.47975	111.794819	1.90E-25	25

Table 2. Bioclim correlation analysis.

		Bio 2	Bio 3	Bio 4	Bio 5	Bio 6	Bio 7	Bio 8	Bio 9	Bio 10	Bio 11	Bio 12	Bio 13	Bio 14	Bio 15	Bio 16	Bio 17	Bio 18	Bio 19
	r	-0.001	0.424	-0.437	**0.839**	**0.918**	-0.392	0.673	0.680	**0.897**	**0.950**	0.026	0.235	-0.294	0.522	0.176	-0.262	0.126	-0.090
Bio 1	r^2	0.000	0.180	0.191	0.704	**0.843**	0.154	0.453	0.462	0.804	**0.902**	0.001	0.055	0.086	0.272	0.031	0.069	0.016	0.008
	VIF	1.000	1.219	1.235	3.380	6.356	1.182	1.827	1.860	5.114	**10.165**	1.001	1.058	1.094	1.374	1.032	1.074	1.016	1.008
	r		0.558	0.080	0.284	-0.203	0.481	0.148	0.165	0.045	-0.019	-0.272	-0.055	-0.410	0.351	-0.029	-0.418	0.029	-0.216
Bio 2	r^2		0.311	0.006	0.081	0.041	0.232	0.022	0.027	0.002	0.000	0.074	0.003	0.168	0.123	0.001	0.175	0.001	0.047
	VIF		1.451	1.006	1.088	1.043	1.302	1.022	1.028	1.002	1.000	1.080	1.003	1.203	1.140	1.001	1.212	1.001	1.049
	r			-0.697	0.234	0.485	-0.383	0.250	0.522	0.135	0.585	-0.029	0.305	-0.420	0.690	0.311	-0.401	0.272	0.027
Bio 3	r^2			0.486	0.055	0.235	0.147	0.062	0.273	0.018	0.342	0.001	0.093	0.177	0.476	0.097	0.161	0.074	0.001
	VIF			1.946	1.058	1.307	1.172	1.067	1.375	1.019	1.519	1.001	1.103	1.214	1.909	1.107	1.192	1.080	1.001
	r				0.065	-0.716	**0.910**	-0.136	-0.520	0.002	-0.693	-0.040	-0.320	0.275	-0.533	-0.300	0.248	-0.224	-0.131
Bio 4	r^2				0.004	0.513	**0.829**	0.019	0.270	0.000	0.480	0.002	0.103	0.076	0.284	0.090	0.061	0.050	0.017
	VIF				1.004	2.052	5.833	1.019	1.370	1.000	1.923	1.002	1.114	1.082	1.396	1.099	1.065	1.053	1.018
	r					0.565	0.165	0.665	0.506	**0.968**	0.654	-0.074	0.061	-0.289	0.372	0.018	-0.272	0.013	-0.210
Bio 5	r^2					0.319	0.027	0.442	0.256	**0.937**	0.427	0.006	0.004	0.084	0.138	0.000	0.074	0.000	0.044
	VIF					1.469	1.028	1.793	1.344	**15.822**	1.746	1.006	1.004	1.091	1.161	1.000	1.080	1.000	1.046
	r						-0.721	0.522	0.690	0.671	**0.981**	0.069	0.297	-0.266	0.538	0.242	-0.232	0.164	0.027
Bio 6	r^2						0.519	0.273	0.475	0.451	**0.963**	0.005	0.088	0.071	0.290	0.059	0.054	0.027	0.001
	VIF						2.081	1.375	1.906	1.820	**26.724**	1.005	1.097	1.076	1.408	1.062	1.057	1.028	1.001
	r							-0.066	-0.399	0.011	-0.624	-0.145	-0.304	0.075	-0.331	-0.275	0.048	-0.186	-0.209
Bio 7	r^2							0.004	0.159	0.000	0.389	0.021	0.092	0.006	0.109	0.075	0.002	0.035	0.044
	VIF							1.004	1.190	1.000	1.636	1.022	1.102	1.006	1.123	1.082	1.002	1.036	1.046
	r								0.231	0.666	0.571	0.147	0.303	-0.165	0.482	0.262	-0.162	0.327	-0.267
Bio 8	r^2								0.053	0.443	0.327	0.022	0.092	0.027	0.232	0.069	0.026	0.107	0.072
	VIF								1.056	1.795	1.485	1.022	1.101	1.028	1.302	1.074	1.027	1.119	1.077
	r									0.522	0.744	-0.258	-0.011	-0.440	0.367	-0.059	-0.386	-0.150	0.144
Bio 9	r^2									0.272	0.553	0.066	0.000	0.194	0.134	0.003	0.149	0.023	0.021
	VIF									1.374	2.239	1.071	1.000	1.241	1.155	1.003	1.176	1.023	1.021
	r										0.719	-0.019	0.082	-0.216	0.321	0.027	-0.194	0.005	-0.159
Bio 10	r^2										0.517	0.000	0.007	0.047	0.103	0.001	0.038	0.000	0.025
	VIF										2.071	1.000	1.007	1.049	1.115	1.001	1.039	1.000	1.026
	r											0.011	0.279	-0.349	0.600	0.226	-0.314	0.157	-0.018
Bio 11	r^2											0.000	0.078	0.122	0.360	0.051	0.099	0.025	0.000
	VIF											1.000	1.085	1.139	1.564	1.054	1.109	1.025	1.000
	r												**0.863**	0.631	0.078	**0.861**	0.668	**0.813**	0.555
Bio 12	r^2												0.745	0.399	0.006	0.741	0.447	0.661	0.308
	VIF												3.918	1.663	1.006	3.859	1.807	2.948	1.445
	r													0.235	0.490	**0.986**	0.275	**0.935**	0.394
Bio 13	r^2													0.055	0.240	**0.971**	0.075	**0.875**	0.155
	VIF													1.058	1.315	**34.986**	1.082	8.000	1.184
	r														-0.557	0.216	**0.983**	0.200	0.483
Bio 14	r^2														0.310	0.047	**0.966**	0.040	0.233
	VIF														1.450	1.049	**29.152**	1.042	1.303
	r															0.505	-0.554	0.473	-0.169
Bio 15	r2															0.255	0.307	0.224	0.029
	VIF															1.342	1.444	1.288	1.030
	r																0.248	**0.950**	0.373
Bio 16	r^2																0.062	**0.902**	0.139
	VIF																1.066	**10.171**	1.162
	r																	0.224	0.550
Bio 17	r^2																	0.050	0.302
	VIF																	1.053	1.434
	r																		0.214
Bio 18	r^2																		0.046
	VIF																		1.048

Table 3a. Algorithm performance using S = 50 average samples per collection variable.

N = Variables in Collection	V = Variables per Run	R = Runs per Sprint	Cores	VMs	Ensembles	Total Runs Needed	C = Cores Available	Cmax = Cores Needed	Total Samples	S = Samples per Variable	T = C Run-time (m)	Tmin = Cmax Run-time (m)	Top Six Variables (bioclim 1st – 6th)						AUC	AICc	Permutation Importance % Top 3	Permutation Importance % Top 4	Number of Viable Predictors
19	2	5	10	10	1	500	100	500	1000	53	6.2	1.18	18	05	03	08	13	16	0.816	12,283	88.1	93.8	5
19	3	3	10	10	1	300	100	300	900	47	5.56	1.96	18	16	13	05	08	12	0.785	12,238	64.5	82.2	4
19	4	3	10	8	1	240	80	240	960	51	6.82	2.25	18	15	13	17	16	14	0.794	12,280	67.4	81.5	4
19	5	2	10	10	1	200	100	200	1000	53	5.78	2.75	18	11	14	15	13	16	0.815	12,142	79.4	91.1	5
19	6	2	10	8	1	160	80	160	960	51	6.67	3.30	18	11	12	15	16	13	0.812	12,174	83.4	95.2	4
19	7	2	10	7	1	140	70	140	980	52	7.81	3.79	18	11	16	13	06	12	0.788	12,179	68.5	80.8	3
19	8	2	10	6	1	120	60	120	960	51	8.47	4.19	18	03	11	13	06	16	0.806	12,303	85.8	92.3	4
19	9	1	10	10	1	100	100	100	900	47	5.16	5.45	18	06	03	17	16	11	0.819	12,155	73.6	86.9	4
19	10	1	10	10	1	100	100	100	1000	53	6.00	5.70	18	06	17	11	12	16	0.813	12,159	72.2	88.5	4
19	11	1	10	9	1	90	90	90	990	52	6.23	5.98	18	03	16	06	17	11	0.801	12,155	79.1	87.2	4
19	12	1	10	8	1	80	80	80	960	51	5.82	5.76	18	17	06	11	15	16	0.817	12,159	73.9	87.4	4
19	13	1	10	7	1	70	70	70	910	48	6.63	6.92	18	11	15	06	03	16	0.815	12,198	61.4	77.6	4
19	14	1	10	7	1	70	70	70	980	52	6.63	6.43	18	03	06	11	12	15	0.819	12,200	68.0	80.0	5
19	15	1	10	6	1	60	60	60	900	47	7.07	7.46	18	03	15	11	06	12	0.803	12,160	91.6	99.9	5
19	16	1	10	6	1	60	60	60	960	51	7.95	7.87	18	06	15	03	12	11	0.815	12,146	68.0	81.2	5
19	17	1	9	6	1	54	54	54	918	48	7.62	7.89	18	06	03	15	11	12	0.817	12,158	65.1	77.6	5
19	18	1	9	6	1	54	54	54	972	51	7.90	7.72	18	06	11	12	09	15	0.823	12,157	65.3	76.2	5
19	19	1	9	5	1	45	45	45	855	45	8.15	9.06	18	15	11	06	10	09	0.817	12,229	75.4	86.9	5
								Max =		53								Max =	0.823	12,303	91.6	99.9	
								Min =		45								Min =	0.785	12,142	61.4	76.2	
								**Avg =**		**50**								**Avg =**	**0.810**	**12,193**	**73.9**	**85.9**	4.4
																		SD =	0.011	50	8.6	6.6	

Parameters set in: MCSprint.R

Parameters set in: mcsprint.sh

Parameters set in: mcensemble.sh

Table 3b. Algorithm performance using S = 100 average samples per collection variable.

N = Variables in Collection	V = Variables per Run	R = Runs per Sprint	Cores	VMs	Ensembles	Total Runs Needed	C = Cores Available	Cmax = Cores Needed	Total Samples	S = Samples per Variable	T = C Run-time (m)	Tmin = Cmax Run-time (m)	Top Six Variables (bioclim 1st – 6th)						AUC	AICc	Permutation Importance % Top 3	Permutation Importance % Top 4	Number of Viable Predictors
19	2	10	10	10	1	1000	100	1000	2000	105	15.03	1.43	18	03	05	13	08	16	0.808	12,241	82.6	90.5	5
19	3	6	10	10	1	600	100	600	1800	95	10.86	1.91	18	08	13	15	14	16	0.805	12,238	64.5	80.1	5
19	4	5	10	10	1	500	100	500	2000	105	11.88	2.26	18	08	13	15	14	16	0.804	12,255	65.5	81.9	5
19	5	4	10	10	1	400	100	400	2000	105	11.33	2.69	18	11	14	15	16	13	0.812	12,152	76.1	87.6	4
19	6	3	10	10	1	300	100	300	1800	95	9.70	3.41	18	06	11	17	16	13	0.815	12,138	70.8	88.9	3
19	7	3	10	9	1	270	90	270	1890	99	10.65	3.57	18	06	17	11	16	13	0.814	12,142	73.0	90.1	3
19	8	3	9	9	1	243	81	243	1944	102	12.23	3.98	18	06	17	11	16	13	0.817	12,149	75.4	92.1	3
19	9	2	10	10	1	200	100	200	1800	95	9.33	4.92	18	03	06	12	11	16	0.808	12,190	76.3	87.9	4
19	10	2	10	10	1	200	100	200	2000	105	12.41	5.89	18	06	17	03	11	16	0.812	12,165	75.6	89.3	4
19	11	2	10	9	1	180	90	180	1980	104	10.39	4.99	18	06	17	03	11	16	0.814	12,171	77.6	92.1	4
19	12	2	10	8	1	160	80	160	1920	101	11.19	5.54	18	11	06	15	03	16	0.819	12,205	67.8	83.0	4
19	13	2	10	7	1	140	70	140	1820	96	12.01	6.27	18	11	06	15	03	16	0.817	12,222	68.7	83.5	4
19	14	2	10	7	1	140	70	140	1960	103	13.08	6.34	18	03	06	11	12	15	0.815	12,146	64.2	77.8	5
19	15	2	10	6	1	120	60	120	1800	95	13.57	7.16	18	11	17	06	15	12	0.821	12,136	74.7	86.6	5
19	16	2	10	6	1	120	60	120	1920	101	14.72	7.28	18	06	12	15	11	09	0.823	12,209	69.0	81.4	5
19	17	2	9	6	1	108	54	108	1836	97	15.08	7.80	18	06	12	11	15	13	0.813	12,166	72.0	84.8	5
19	18	2	9	6	1	108	54	108	1944	102	15.89	7.77	18	06	15	12	09	11	0.823	12,158	66.8	81.0	5
19	19	2	9	6	1	108	54	108	2052	108	16.52	7.65	18	06	10	12	15	11	0.821	12,116	65.7	78.9	5
Parameters set in: MCSprint.R									Max =	108								Max =	0.823	12,255	77.6	92.1	
Parameters set in: mcsprint.sh									Min =	95								Min =	0.804	12,116	64.2	77.8	
Parameters set in: mcensemble.sh									**Avg =**	**101**								**Avg =**	**0.815**	**12,178**	**70.8**	**85.1**	4.3
																		SD =	0.006	40	5.1	4.5	

Table 3c. Algorithm performance using S = 150 average samples per collection variable.

N = Variables in Collection	V = Variables per Run	R = Runs per Sprint	Cores	VMs	Ensembles	Total Runs Needed	C = Cores Available	Cmax = Cores Needed	Total Samples	S = Samples per Variable	T = C Run-time (m)	Tmin = Cmax Run-time (m)	Top Six Variables (bioclim 1st – 6th)						AUC	AICc	Permutation Importance % Top 3	Permutation Importance % Top 4	Number of Viable Predictors
19	2	14	10	10	1	1400	100	1400	2800	147	17.56	1.28	18	16	12	05	13	08	0.798	12,247	66.9	79.8	4
19	3	10	10	10	1	1000	100	1000	3000	158	16.82	1.60	18	15	08	14	13	16	0.803	12,225	63.8	79.7	5
19	4	7	10	10	1	700	100	700	2800	147	15.05	2.19	18	08	15	14	13	16	0.801	12,235	68.3	83.1	5
19	5	6	10	10	1	600	100	600	3000	158	15.28	2.42	18	13	15	11	16	12	0.785	12,174	81.6	95.6	5
19	6	5	10	10	1	500	100	500	3000	158	15.38	2.92	18	06	14	11	16	13	0.812	12,171	69.6	85.4	4
19	7	4	10	10	1	400	100	400	2800	147	14.86	3.78	18	16	17	13	11	06	0.797	12,151	66.4	82.6	4
19	8	4	10	9	1	360	90	360	2880	152	16.03	3.97	18	06	17	11	16	13	0.815	12,167	69.0	88.3	4
19	9	4	10	8	1	320	80	320	2880	152	17.00	4.21	18	06	17	11	12	16	0.814	12,152	73.8	91.3	4
19	10	4	10	7	1	280	70	280	2800	147	19.65	5.00	18	06	17	03	11	16	0.815	12,165	78.5	90.7	4
19	11	4	9	7	1	252	63	252	2772	146	21.90	5.63	18	17	06	03	11	16	0.815	12,154	75.6	92.1	4
19	12	3	10	8	1	240	80	240	2880	152	16.75	5.53	18	06	03	17	11	16	0.817	12,155	77.8	91.2	4
19	13	3	9	8	1	216	72	216	2808	148	17.60	5.95	18	06	15	12	11	16	0.817	12,172	70.0	83.4	4
19	14	3	10	7	1	210	70	210	2940	155	19.88	6.42	18	11	06	03	15	16	0.815	12,203	67.6	82.7	4
19	15	3	10	6	1	180	60	180	2700	142	21.70	7.64	18	11	06	15	03	12	0.819	12,161	65.8	79.3	5
19	16	3	10	6	1	180	60	180	2880	152	21.55	7.11	18	06	11	15	12	03	0.813	12,149	65.1	76.2	5
19	17	2	10	8	1	160	80	160	2720	143	15.70	8.23	18	06	12	11	09	15	0.820	12,151	68.1	79.3	5
19	18	2	10	8	1	160	80	160	2880	152	16.50	8.16	18	11	06	03	15	09	0.817	12,240	75.3	86.8	5
19	19	2	9	8	1	144	72	144	2736	144	16.95	8.83	18	06	12	11	15	09	0.825	12,194	65.6	78.1	5
									Max =	158								Max =	0.825	12,247	81.6	95.6	
									Min =	142								Min =	0.785	12,149	63.8	76.2	
									**Avg =**	**150**								**Avg =**	**0.811**	**12,181**	**70.5**	**84.8**	4.4
																		SD =	0.010	33	5.1	5.5	

Parameters set in: MCSprint.R
Parameters set in: mcsprint.sh
Parameters set in: mcensemble.sh

Table 4. Contribution analysis.

		Bio 2		Bio 3		Bio 4		Bio 5		Bio 6		Bio 7		Bio 8		Bio 9		Bio 10		Bio 11		Bio 12	
	PI<br>PC<br>JK		31.3<br>19.6<br>0.019		55.7<br>59.7<br>0.120		48.3<br>52.2<br>0.118		86.4<br>94.4<br>0.222		36.7<br>23.1<br>0.101		28.3<br>11.8<br>0.015		88.5<br>97.4<br>0.325		60.2<br>52.7<br>0.191		68.8<br>87.8<br>0.161		**54.1**<br>45.8<br>0.150		59.9<br>66.7<br>0.244
Bio 1	PI<br>PC<br>JK	68.7<br>80.4<br>0.126		44.3<br>40.3<br>0.120		**51.7**<br>47.8<br>0.125		13.6<br>5.6<br>0.116		63.3<br>76.9<br>0.114		71.7<br>88.2<br>0.117		11.5<br>2.6<br>0.116		39.8<br>47.3<br>0.118		31.2<br>12.2<br>0.114		45.9<br>54.2<br>0.120		40.1<br>33.3<br>0.121	
	PI<br>PC<br>JK				86.9<br>91.1<br>0.166		67.7<br>81.1<br>0.115		90.3<br>95.8<br>0.225		59.2<br>75.4<br>0.105		47.9<br>45.1<br>0.015		90.5<br>96.1<br>0.321		84.7<br>90.3<br>0.191		85.0<br>92.9<br>0.161		67.5<br>80.5<br>0.148		79.5<br>93.3<br>0.250
Bio 2	PI<br>PC<br>JK			13.1<br>8.9<br>0.019		32.3<br>18.9<br>0.018		9.7<br>4.2<br>0.019		40.8<br>24.6<br>0.022		52.1<br>54.9<br>0.019		9.5<br>3.9<br>0.015		15.3<br>9.7<br>0.020		15.0<br>7.1<br>0.021		32.5<br>19.5<br>0.019		20.5<br>6.7<br>0.022	
	PI<br>PC<br>JK						34.0<br>16.6<br>0.116		57.9<br>52.0<br>0.223		41.1<br>37.6<br>0.103		8.6<br>4.3<br>0.016		60.4<br>59.5<br>0.322		53.1<br>54.9<br>0.191		45.5<br>46.1<br>0.165		43.6<br>40.8<br>0.156		60.4<br>59.7<br>0.247
Bio 3	PI<br>PC<br>JK					66.0<br>83.4<br>0.170		42.1<br>48.0<br>0.170		58.9<br>62.4<br>0.163		91.4<br>95.7<br>0.176		39.6<br>40.5<br>0.177		46.9<br>45.1<br>0.169		54.5<br>53.9<br>0.172		56.4<br>59.2<br>0.170		39.6<br>40.3<br>0.159	
	PI<br>PC<br>JK								60.9<br>59.1<br>0.217		44.4<br>46.3<br>0.105		26.3<br>15.7<br>0.015		72.4<br>71.9<br>0.324		56.1<br>60.0<br>0.190		54.6<br>54.4<br>0.162		**53.0**<br>49.8<br>0.149		61.4<br>67.0<br>0.240
Bio 4	PI<br>PC<br>JK							39.1<br>40.9<br>0.125		55.6<br>53.7<br>0.121		73.7<br>84.3<br>0.118		27.6<br>28.1<br>0.110		43.9<br>40.0<br>0.122		45.4<br>45.6<br>0.121		47.0<br>50.2<br>0.119		38.6<br>33.0<br>0.115	
	PI<br>PC<br>JK										19.7<br>10.2<br>0.103		13.5<br>8.6<br>0.017		73.4<br>87.8<br>0.314		53.5<br>49.8<br>0.202		7.5<br>1.4<br>0.164		25.6<br>19.3<br>0.143		48.1<br>59.7<br>0.254
Bio 5	PI<br>PC<br>JK									80.3<br>89.8<br>0.229		86.5<br>91.4<br>0.228		26.6<br>12.2<br>0.214		46.5<br>50.2<br>0.226		92.5<br>98.6<br>0.228		74.4<br>80.7<br>0.219		**51.9**<br>40.3<br>0.224	
	PI<br>PC<br>JK												25.1<br>16.9<br>0.016		89.7<br>95.5<br>0.310		61.6<br>55.6<br>0.196		62.1<br>82.2<br>0.162		59.4<br>80.5<br>0.152		58.2<br>69.8<br>0.247
Bio 6	PI<br>PC<br>JK											74.9<br>83.1<br>0.101		10.3<br>4.5<br>0.101		38.4<br>44.4<br>0.108		37.9<br>17.8<br>0.105		40.6<br>19.5<br>0.103		41.8<br>30.2<br>0.101	
	PI<br>PC<br>JK														89.9<br>96.6<br>0.322		93.8<br>97.5<br>0.191		78.7<br>89.5<br>0.159		92.9<br>97.9<br>0.155		79.3<br>93.4<br>0.240
Bio 7	PI<br>PC<br>JK													10.1<br>3.4<br>0.014		6.2<br>2.5<br>0.016		21.3<br>10.5<br>0.013		7.1<br>2.1<br>0.016		20.7<br>6.6<br>0.015	
	PI<br>PC<br>JK																33.3<br>27.8<br>0.194		24.1<br>8.5<br>0.159		23.7<br>13.1<br>0.154		37.6<br>49.5<br>0.250
Bio 8	PI<br>PC<br>JK															66.7<br>72.2<br>0.330		75.9<br>91.5<br>0.318		76.3<br>86.9<br>0.327		62.4<br>50.5<br>0.322	
	PI<br>PC<br>JK																		34.2<br>44.8<br>0.157		47.4<br>46.3<br>0.146		56.5<br>68.1<br>0.247
Bio 9	PI<br>PC<br>JK																	65.8<br>55.2<br>0.202		52.6<br>53.7<br>0.192		43.5<br>31.9<br>0.199	
	PI<br>PC<br>JK																				44.1<br>37.0<br>0.151		58.3<br>64.5<br>0.246
Bio 10	PI<br>PC<br>JK																			55.9<br>63.0<br>0.153		41.7<br>35.5<br>0.161	
	PI<br>PC<br>JK																						58.3<br>62.6<br>0.239
Bio 11	PI<br>PC<br>JK																					41.7<br>37.4<br>0.148	
	PI<br>PC<br>JK																						
Bio 12	PI<br>PC<br>JK																						
	PI<br>PC<br>JK																						
Bio 13	PI<br>PC<br>JK																						
	PI<br>PC<br>JK																						
Bio 14	PI<br>PC<br>JK																						
	PI<br>PC<br>JK																						
Bio 15	PI<br>PC<br>JK																						
	PI<br>PC<br>JK																						
Bio 16	PI<br>PC<br>JK																						
	PI<br>PC<br>JK																						
Bio 17	PI<br>PC<br>JK																						
	PI<br>PC<br>JK																						
Bio 18	PI<br>PC<br>JK																						

PI = Permutation Importance, PC = Percent Contribution, JK = Jackknife Analysis (training gain) / Blue background = Non-corresponding PI / Yellow background = Correlated variables from Table 2

Table 4. Contribution analysis, continued.

		Bio 13		Bio 14		Bio 15		Bio 16		Bio 17		Bio 18		Bio 19	
	PI		76.6		51.9		61.1		80.5		51.7		79.6		36.8
	PC		82.7		59.0		80.1		84.4		59.7		85.3		33.8
	JK		0.388		0.172		0.261		0.427		0.165		0.477		0.020
Bio 1	PI	23.4		48.1		38.9		19.5		48.3		20.4		63.2	
	PC	17.3		41.0		19.9		15.6		40.3		14.7		66.2	
	JK	0.119		0.120		0.127		0.120		0.112		0.122		0.117	
	PI		90.9		65.3		74.6		91.3		65.6		94.5		54.8
	PC		96.7		91.1		92.8		97.6		92.5		98.9		55.5
	JK		0.389		0.172		0.249		0.424		0.152		0.484		0.019
Bio 2	PI	9.1		34.7		25.4		8.7		34.4		5.5		45.2	
	PC	3.3		8.9		7.2		2.4		7.5		1.1		44.5	
	JK	0.019		0.022		0.019		0.020		0.018		0.020		0.015	
	PI		63.2		53.5		45.5		68.2		**51.6**		73.1		22.3
	PC		62.0		60.1		53.3		63.2		55.7		70.9		10.1
	JK		0.387		0.177		0.240		0.427		0.165		0.485		0.018
Bio 3	PI	36.8		46.5		**54.5**		31.8		48.4		26.9		77.7	
	PC	38.0		39.9		46.7		36.8		44.3		29.1		89.9	
	JK	0.168		0.172		0.163		0.170		0.175		0.167		0.169	
	PI		74.3		58.3		61.5		76.8		53.0		82.9		33.0
	PC		73.7		68.5		69.1		75.6		64.2		84.5		18.8
	JK		0.396		0.163		0.245		0.425		0.152		0.486		0.019
Bio 4	PI	25.7		41.7		38.5		23.2		47.0		17.1		67.0	
	PC	26.3		31.5		30.9		24.4		35.8		15.5		81.2	
	JK	0.115		0.109		0.118		0.116		0.118		0.118		0.124	
	PI		66.0		46.2		55.1		77.5		43.6		76.6		8.4
	PC		76.6		52.8		65.0		79.0		49.8		82.0		3.9
	JK		0.395		0.177		0.253		0.428		0.166		0.475		0.020
Bio 5	PI	34.0		**53.8**		44.9		22.5		56.4		23.4		91.6	
	PC	23.4		47.2		35.0		21.0		50.2		18.0		96.1	
	JK	0.229		0.222		0.216		0.227		0.225		0.225		0.223	
	PI		73.7		53.4		52.3		80.8		52.1		85.9		41.3
	PC		85.0		57.7		72.7		86.5		53.7		87.6		26.3
	JK		0.387		0.169		0.246		0.428		0.156		0.475		0.018
Bio 6	PI	26.3		46.6		47.7		19.2		47.9		14.1		58.7	
	PC	15.0		42.3		27.3		13.5		46.3		12.4		73.7	
	JK	0.099		0.101		0.104		0.101		0.107		0.105		0.103	
	PI		87.6		61.2		69.2		89.5		59.3		94.4		50.0
	PC		96.1		90.0		88.7		96.7		89.0		98.8		55.0
	JK		0.393		0.168		0.251		0.428		0.155		0.486		0.020
Bio 7	PI	12.4		38.8		30.8		10.5		40.7		5.6		50.0	
	PC	3.9		10.0		11.3		3.3		11.0		1.2		45.0	
	JK	0.015		0.015		0.015		0.016		0.014		0.015		0.019	
	PI		60.4		35.2		39.0		65.2		32.2		65.9		7.8
	PC		72.0		40.7		41.5		75.4		36.4		81.1		2.1
	JK		0.393		0.165		0.252		0.434		0.155		0.482		0.020
Bio 8	PI	39.6		64.8		61.0		34.8		67.8		34.1		92.2	
	PC	28.0		59.3		58.5		24.6		63.6		18.9		97.9	
	JK	0.324		0.333		0.329		0.317		0.331		0.321		0.331	
	PI		72.9		53.2		56.5		75.0		47.2		84.2		30.8
	PC		83.2		62.3		59.1		86.8		57.1		89.1		15.6
	JK		0.394		0.169		0.250		0.418		0.158		0.477		0.018
Bio 9	PI	27.1		46.8		43.5		25.0		**52.8**		15.8		69.2	
	PC	16.8		37.7		40.9		13.2		42.9		10.9		84.4	
	JK	0.196		0.190		0.196		0.188		0.189		0.198		0.191	
	PI		74.7		52.8		59.4		78.9		58.0		83.9		26.3
	PC		79.4		54.4		70.5		82.7		56.7		85.0		12.3
	JK		0.377		0.166		0.250		0.426		0.166		0.478		0.020
Bio 10	PI	25.3		47.2		40.6		21.1		42.0		16.1		73.7	
	PC	20.6		45.6		29.5		17.3		43.3		15.0		87.7	
	JK	0.162		0.159		0.167		0.151		0.157		0.160		0.161	
	PI		67.6		47.1		50.2		78.1		45.7		80.4		39.8
	PC		80.5		58.2		71.2		81.3		56.3		84.3		27.4
	JK		0.379		0.163		0.250		0.422		0.156		0.476		0.022
Bio 11	PI	32.4		**52.9**		49.8		21.9		**54.3**		19.6		60.2	
	PC	19.5		41.8		28.8		18.7		43.7		15.7		72.6	
	JK	0.140		0.150		0.155		0.152		0.153		0.149		0.154	
	PI		85.2		39.4		51.0		80.7		41.2		86.0		26.7
	PC		97.1		40.0		51.3		94.4		31.3		95.3		14.4
	JK		0.393		0.168		0.257		0.431		0.157		0.492		0.020
Bio 12	PI	14.8		60.6		49.0		19.3		58.8		14.0		73.3	
	PC	2.9		60.0		48.7		5.6		68.7		4.7		85.6	
	JK	0.245		0.255		0.259		0.251		0.247		0.247		0.252	
	PI				29.1		37.8		46.3		25.6		64.4		18.2
	PC				23.5		35.8		28.6		21.3		75.3		8.1
	JK				0.180		0.245		0.452		0.166		0.496		0.022
Bio 13	PI			70.9		62.2		53.7		74.4		35.6		81.8	
	PC			76.5		64.2		71.4		78.7		24.7		91.9	
	JK			0.408		0.364		0.414		0.395		0.401		0.419	
	PI						53.5		75.9		24.9		80.9		31.2
	PC						53.5		79.4		8.8		83.7		15.0
	JK						0.248		0.448		0.163		0.511		0.018
Bio 14	PI					46.5		24.1		75.1		19.1		68.8	
	PC					46.5		20.6		91.2		16.3		85.0	
	JK					0.168		0.177		0.172		0.181		0.178	
	PI								69.2		40.7		72.9		22.1
	PC								66.4		44.0		70.7		8.6
	JK								0.427		0.162		0.478		0.022
Bio 15	PI							30.8		59.3		27.1		77.9	
	PC							33.6		56.0		29.3		91.4	
	JK							0.249		0.248		0.254		0.242	
	PI										25.1		61.4		12.9
	PC										17.9		79.7		6.6
	JK										0.161		0.502		0.021
Bio 16	PI									74.9		38.6		87.1	
	PC									82.1		20.3		93.4	
	JK									0.438		0.446		0.432	
	PI												82.0		38.7
	PC												86.3		23.6
	JK												0.495		0.024
Bio 17	PI											18.0		61.3	
	PC											13.7		76.4	
	JK											0.166		0.166	
	PI														4.7
	PC														1.2
	JK														0.020
Bio 18	PI													95.3	
	PC													98.8	
	JK													0.501	

References

1. Muscarella R, Galante PJ, Soley-Guardia M, Boria RA, Kass JM, Uriarte M, et al. ENMeval: An R package for conducting spatially independent evaluations and estimating optimal model complexity for Maxent ecological niche models. Methods in Ecology and Evolution. 2014;5: 1198–1205. doi:10.1111/2041-210X.12261

2. Muscarella R, Galante PJ, Soley-Guardia M, Boria RA, Kass JM, Anderson MU and RP. ENMeval: Automated Runs and Evaluations of Ecological Niche Models. 2018. Available: https://CRAN.R-project.org/package=ENMeval

3. Pradhan P. Strengthening MaxEnt modelling through screening of redundant explanatory bioclimatic variables with variance inflation factor analysis. Researcher. 2016;8: 29–34.

4. Phillips SJ. A Brief Tutorial on Maxent. AT&T Research. 2005;190: 231–259.

5. Smith AB, Santos MJ. Testing the ability of species distribution models to infer variable importance. Ecography. 2020;43: 1801–1813. doi:10.1111/ecog.05317

6. Phillips SJ, Dudík M. Modeling of species distributions with Maxent: new extensions and a comprehensive evaluation. Ecography. 2008;31: 161–175.

7. Cobos ME, Peterson AT, Osorio-Olvera L, Jiménez-García D. An exhaustive analysis of heuristic methods for variable selection in ecological niche modeling and species distribution modeling. Ecological Informatics. 2019;53: 100983. doi:10.1016/j.ecoinf.2019.100983
